# Supplementary material for: Comparison of volumetric brain analysis in subjects with rheumatoid arthritis and ulcerative colitis
Source: Front Med (Lausanne). 2024 Nov 20;11:1468910. doi: 10.3389/fmed.2024.1468910 (PMC11614619; doi:10.3389/fmed.2024.1468910)
Supplement: Supplementary file 1 [file Data_Sheet_1.PDF]

Supplementary Materials for “Comparison of volumetric brain analysis in subjects with rheumatoid arthritis and ulcerative colitis”

Table 5: Model 2 for hippocampal volume in RA and UC includes gender, age, ICV and hypertension as covariates

	Region of Interest	p-value	Mean Volume Patient Population (mL)	Mean Volume Control Population (mL)	Cohen’s d	95% CI
RA	Left Hippocampus	0.84	3.7+/- 0.5	3.7 +/- 0.4	-0.04	-0.18, 0.1
	Right Hippocampus	0.36	3.8 +/- 0.5	3.8 +/- 0.5	-0.08	-0.22, 0.06
	Total Hippocampus	0.52	7.5 +/- 0.9	7.5 +/- 0.8	-0.07	-0.21, 0.07
UC	Left Hippocampus	0.20	3.7 +/- 0.5	3.8 +/- 0.5	-0.13	-0.27, 0.02
	Right Hippocampus	0.08	3.8 +/- 0.5	3.9 +/- 0.5	-0.15	-0.3, -0.01
	Total Hippocampus	0.08	7.6 +/- 0.9	7.7 +/- 0.8	-0.16	-0.3, -0.01
